# Including genotypic information in genetic evaluations increases the accuracy of sheep breeding values

**DOI:** 10.1111/jbg.12771

**Published:** 2023-04-01

**Authors:** Karolina Kaseja, Sebastian Mucha, Ed Smith, John Yates, Georgios Banos, Joanne Conington

**Affiliations:** ^1^ Scotland's Rural College (SRUC) Easter Bush, Roslin Institute Building Edinburgh EH25 9RG UK; ^2^ The British Texel Sheep Society Stoneleigh Park Warwickshire CV8 2LG UK

**Keywords:** accuracy change, animal welfare, breeding values, genetic parameters, genomic selection, meat sheep, sheep breeding, single‐step

## Abstract

The impact of inclusion of genome‐wide genotypes into breeding value predictions for UK Texel sheep is addressed in this article. The main aim was to investigate the level of change in the accuracy values for EBVs when information from animal genotypes is incorporated into the genetic evaluations. New genetic parameters for a range of lamb growth, carcass composition and health traits are described and applied in the estimation of conventional breeding values (EBVs) for almost 822,000 animals as well as genomic breeding values (gEBVs) after adding 10,143 genotypes. Principal component analyses showed that there are no major distinct groups; hence, the population is mainly homogenous and genetically well‐linked. Results suggested that the highest change in accuracy was observed for the animals that are not phenotyped but have good links to the reference population. This was seen especially for the lowly heritable health traits thereby proving that the use of genotypes in breeding values estimation may accelerate the genetic gain by producing more accurate values especially for young, un‐phenotyped animals.

**Implications**

The use of animal genotypes from genome‐wide DNA arrays in the genetic evaluation process has become a new standard for many livestock species. Improving the accuracy of breeding value estimates is critical to the success of breeding programmes. Accuracies are often low especially for lowly heritable traits with low numbers of phenotypic measurements. They are also often low when the traits of interest are expressed in female adults, but males are selected as young stock, such as in sheep breeding programmes. This research shows that incorporating genomic information into the genetic evaluation increases the accuracies of breeding values enabling selection especially for animals without phenotypes and for low to moderate heritability traits. It allows for more accurate selection of males and female replacements to be made at an earlier stage in life without having to wait for phenotypes from adult females to be collected.

## 1 INTRODUCTION

Genomic evaluation is now widely used as a breeding tool for genetic selection in several species of farm animals across the world but is less well developed for ovine (Hayes et al., 2012; Berry et al., 2016, Rupp et al., 2016, Fitzmaurice et al., 2021, Berry et al., 2022). In the UK, estimation of breeding values in sheep is based only on the conventional Best Linear Unbiased Prediction (BLUP, Henderson, 1949) method of analysis of animal phenotypic and pedigree data provided by the breeders and Breed Societies. This approach works well, especially for animal traits with moderate to high heritability and/or sufficient amounts of phenotypic data available. Availability of informative pedigree linking animals from different flocks is also a prerequisite (Simm, 1998). These conditions are met for multiple growth and carcass traits; however, this method has a limitation to the maximum accuracy level of the estimated breeding values (EBV) that can be achieved by young animals which have not yet had the chance to be phenotyped. Furthermore, for traits that are difficult to measure, measured on one sex only or with low heritability, such as reproduction and health traits, the accuracy of EBV may be low, even for animals with measured phenotypes. This means that selection decisions based on such EBVs may slow down the achievement of the genetic goal set by the breeders. The alternative approach, and one which simultaneously combines animal genotypes with phenotypes and pedigree, is Single‐Step BLUP (SS‐BLUP) (Christensen & Lund, 2010; Legarra et al., 2009; Misztal et al., 2009). This approach may lead to more accurate EBVs and reduce the generation interval by enabling an early selection of candidates for selection, especially for traits that can be measured late in life or on adult progeny only, such as mastitis.

Genomic selection is already used in small ruminant breeding in many countries, such as Australia (https://www.sheepgenetics.org.au), New Zealand (Auvray et al., 2014), Ireland (https://www.sheep.ie/), France (Palhière et al., 2022) or internationally (Teissier et al., 2022) mostly addressing production traits. Furthermore, incorporating health traits such as mastitis or parasite resistance to the breeding programmes might affect positively the overall animal welfare, as well as the economic gain (Pacheco et al., 2021; Walkom et al., 2022), and this will be possible only if the accuracy of animal EBVs is satisfactorily high and above a certain threshold, which would allow publication of EBVs and reduce risks associated with making selection decisions. Minimum accuracy thresholds are extensively used across variety of traits in many species in the UK (www.fas.scot), Australia (https://breedplan.une.edu.au/general/understanding‐ebv‐accuracy/) and Ireland (Sheep Ireland Guide & Directory of Breeders, 2020).

The objective of the present study was to assess the impact of incorporating genomic information in the EBV estimation on the accuracy of genetic evaluation for health (footrot and mastitis) and production (birth weight, weaning weight, scan weight and fat and muscle depth) traits in UK Texel sheep.

## 2 MATERIALS AND METHODS

### 2.1 Phenotypes

Two sets of traits were examined in this research. Firstly, these included growth and carcass composition traits measured in lambs, which were: birth weight (BWT), eight‐week weight (EWW, growth rate to 8 weeks of age), scan weight (SWT, growth rate to finishing), muscle depth (MD, loin muscularity) and fat depth (FD, potential to produce lean/fat carcasses). Growth and carcass measurements of sheep were taken from the iTexel database, where data for these phenotypes are routinely reported by the breeders. This dataset contained phenotypes for 645,840 animals born between 1970 and 2021.

Secondly, we included health traits measured in adult ewes, namely, footrot (FRT) and California Mastitis Test score (CMT). The CMT was used instead of the conventional somatic cell count in milk as a mastitis indicator because it was found to be highly (up to 0.98) correlated with somatic cell count (McLaren et al., 2018), and can be measured on‐farm without the need for laboratory analyses. For health traits (FRT and CMT), the data were collected specifically for this research project between 2015 and 2019 on 32 farms across the UK on 3434 milking females. Trained technicians visited the farms at least once per year to score animals using a five‐point scale, from 0 – not infected – to 4 – severe infection (Conington et al., 2008; McLaren et al., 2018) – for both health traits. Animals were scored between one and five times over the course of 5 years in 2015–2019.

Further edits on phenotypes were performed, removing values that were out of biologically expected ranges, as summarized in Table 1.

**TABLE 1:** Data description by trait.

Trait (unit of measurement)	Range of biologically accepted values	No. animals with phenotypic records used for breeding value estimation	No. animals with phenotypic records used for parameter estimation	No. animals with genotype and phenotype
Birth weight (kg)	2–10	188,606	33,857	2168
Eight‐week weight (kg)	5–50	402,787	72,845	5087
Scan weight (kg)	10–150	296,239	70,492	5608
Muscle depth (mm)	5–55	284,317	67,865	5513
Fat depth (mm)	0.1–20	283,644	67,867	5512
Footrot (0–4 scale; severity of infection)	0 (no infection) – 4 (severe infection)	6216	6216	3778
California mastitis Test (0–4 scale; severity of infection)	0 (no infection) – 4 (severe infection)	3346	3346	2909

### 2.2 Genotypes

The data used in this research contain 10,193 Texel sheep genotypes, collected between January 2015 and March 2019 on participating partner farms in the UK from male and female animals. Animals were genotyped with four SNP arrays, including 1180 animals genotyped on the Illumina OvineHD BeadChip with 606,006 SNPs (HD), 2894 animals genotyped on Illumina OvineSNP50 with 54,241 SNPs (50 K), 2463 animals genotyped on Illumina OvineLD BeadChip with 15,000 SNPs (LDv1) and 3606 animals genotyped on Illumina OvineLD BeadChip with 16,560 SNPs (LDv2).

Animal genotypic data received from the genotyping laboratory were subjected to a series of quality control procedures. These included the rejection of genotypes that did not meet the call rate threshold of 89.4% (which is used in the UK national genomic evaluations for cattle). Subsequently, a parentage check was undertaken to discard genotypes for which the parentage was not confirmed. The opposing homozygotes method (Hayes, 2011) was used on a subset of 8119 SNPs that were common to the four genotyping arrays as described in Kaseja et al. (2022), and if an animal failed the genomic parentage verification (over 1% of conflicting SNPs) and the correct parent did not appear from the parentage discovery, the unverified parent was set to “unknown.” Additionally, when there was more than one sample per animal and only one passed parentage verification, then that sample was kept. If more than one sample confirmed the parentage, then the genotypes were compared with each other to confirm they were identical, and if so, one genotype was chosen based on the density of the SNP array used for genotyping, in the following order of priority: 50 K, HD, LDv2 and LDv1.

The final dataset contained 9391 genotypes (971 HD, 2709 50 K, 2350 LDv1 and 3361 LDv2). The next stage was quality control checks at the SNP level, which involved removing SNPs with call rate under 89.4%, minor allele frequency below 0.05 and not in Hardy–Weinberg equilibrium (*p*‐value at 0.05), thereby producing subsets of 450,686, 36,654, 12,427 and 10,725 SNPs for HD, 50 K, LDv2 and LDv1 SNP arrays, respectively. All genotypes were then imputed to the subset of most informative SNPs from the 50 K array (*n* = 36,654 SNPs) using Findhap V3 software (VanRaden et al., 2011).

The population structure to elucidate breed composition was determined using Principal Component Analysis (PCA) (Macciotta et al., 2010; Mucha, Bunger, & Conington, 2015; Mucha, Mrode, et al., 2015) in R software (R Core Team, 2022).

### 2.3 Pedigree

A pedigree file including all phenotyped animals and their parents (*n* = 821,692) was built using information provided by the breeders on the iTexel database and altered accordingly based on the information from genomic parentage verification and discovery as described above (where possible).

### 2.4 Data analysis

The following mixed effect model was used in all data analyses:

(1) y=Xb+Za+Wp+e

where y is vector of observations, X is design matrix of order, relating records to b – vector of fixed effects, Z is design matrix of order, relating records to a – vector of random additive genetic effects, W is design matrix for random permanent environment effect, p is vector for random permanent environment effect, and e is a vector of random residual effects. Random effects were assumed to be normally distributed with the mean of zero. A summary of the fixed and random effects is shown in Table 2. As the data were collected across many years and in many flocks, contemporary grouping (CG) was used to compare animals more directly. CG were defined as flock‐season of birth‐sex for production traits or month‐year and farm‐year for CMT.

**TABLE 2:** Fixed and random effects considered in parameter and breeding value estimation.

Trait	Fixed effects	Random effects
Birth weight	LSB DamAge DamBreed ET CG	Animal Dam PE
Eight‐week weight	LSB DamAge DamBreed Foster CG	Animal Dam PE
Scan weight	LSR DamAge DamBreed CG covariable: age at scan	Animal
Muscle depth	LSR DamAge DamBreed CG Adjustment: age at scan	Animal
Fat depth	LSR DamAge DamBreed CG Adjustment: age at scan	Animal
Footrot	DamAge Scorer Vax Flock	Animal
California Mastitis Test	Lambing LSB Scorer CG2	Animal PE

Abbreviations: CG, contemporary group as flock‐season‐sex; CG2, contemporary group as month‐year and farm‐year; ET, embryo transfer status; Foster, foster code status; LSB, litter size born; PE, permanent environment.; Vax, vaccination status for footrot.

Both FRT and CMT were analysed as the natural logarithm of the sum of scores for all hooves and both udder halves, respectively, plus one (to avoid sum of zero) as described in McLaren et al. (2018).

### 2.5 Parameter estimation

Variance components were first estimated for each trait with Model (1) using the ASReml software (Gilmour et al., 2015), with the use of an informative subset of data containing only animals with at least one valid phenotype, born between 2011 and 2021. Additional data edits in this step excluded lambs that had been fostered, were born as a result of embryo transfer or were born within a litter of over four lambs. The contemporary group had to have a minimum of five individuals. The data used for parameter estimations are summarized in Table 1. In this analysis, the random additive genetic effects were assumed to be normally distributed ~ $\left(0,{\mathrm{A\sigma}}_{a}^{2}\right)$ for all animals, where $\sigma_{a}^{2}$ is the additive genetic variance and A is the pedigree relationship matrix. A separate series of bivariate analyses based on Model (1)‐derived estimates of the genetic and phenotypic correlations among the studied traits.

The estimated variance components were then used to derive animal EBVs for each trait with Model (1) and the MiX99 software (Lidauer et al., 2015).

### 2.6 Genomic prediction

Conventional BLUP estimates were derived first with the same distribution assumptions for the random effects as the variance component estimation step. Subsequently, the random genetic effect variance structure was modified to accommodate inclusion information from two sources: G^−1^‐A^−1^_gg_ where G (obtained using first method from VanRaden, 2008) is a genomic relationship matrix and A_gg_ is pedigree‐based relationship matrix for genotyped animals, replacing in the model the A matrix with H – combined relationship matrix (Christensen, 2012). The key difference between these two analyses was the addition of information from animals' genotypes to SS‐BLUP, while pedigree and phenotypes remained the same in both.

Breeding values were estimated for the full available dataset (*n* = 821,692 animals). Reliabilities of the estimated breeding values were estimated using Apax99 software (Lidauer et al., 2015), using a two‐step method which, in the first instance, calculates information due to observations coming from the above model and secondly uses Misztal and Wiggans (1988) method to add the relationship information. The reliabilities produced were subsequently converted to accuracy values by using their square root value.

## 3 RESULTS AND DISCUSSION

### 3.1 Population structure

**FIGURE 1:**
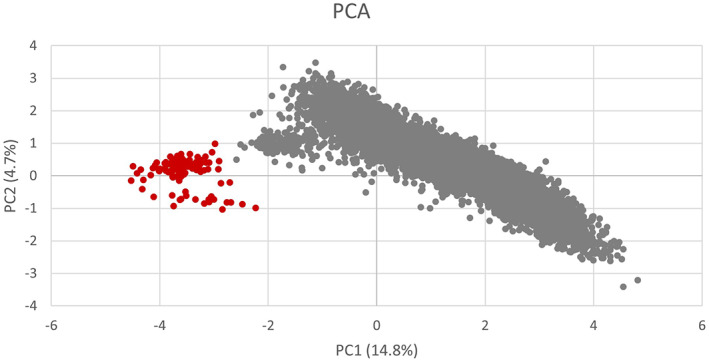
Plot of first (PC1) and second (PC2) principal component of the genomic relationship matrix for all genotyped animals. Animals originated from New Zealand coloured in red. [Colour figure can be viewed at wileyonlinelibrary.com]

The results from the PCA to determine population structure are shown in Figure 1. Clustering based on the principal components of the genotype matrix did not reveal any major outliers, indicating that the population is mostly homogenous. The first two principal components explained 14.8% and 4.7% of variation, respectively. The obtained structure of this population is showing a small cluster of 80 animals being somewhat separated from the main cluster. Further investigation has indicated that these animals were imported into the UK from New Zealand; hence, their genetic background differs from the rest of the main population which is UK Texel sheep.

### 3.2 Genetic parameters

A summary of variance components and genetic parameters by trait is shown in Table 3. The trait heritabilities range from 0.07 (low, for CMT) to 0.33 (moderate, for SWT) and are in line with the heritabilities obtained from similar research for growth and health traits in sheep (McLaren et al., 2018; Mucha, Bunger, & Conington, 2015; Mucha, Mrode, et al., 2015; Safari et al., 2005). All correlations between traits estimated using the bivariate models are summarized in Table 4. The genetic correlation between FRT and CMT was 0.28 (±0.11), indicating likely pleiotropic effects on these two health traits. To the authors' knowledge, these are the first estimates of mastitis and lameness correlation for meat sheep. The genetic correlations between the health traits and growth or body composition traits were not significantly different from zero. However, the correlations estimated among lamb growth and body composition were significantly different from zero and within the range of values expected and previously reported for example by Fitzmaurice et al. (2021), Lambe et al. (2008) or Mortimer et al. (2018).

**TABLE 3:** Estimated variance components and parameters, followed by standard errors.

Trait	Direct genetic variance	Permanent environment variance	Maternal variance	Residual variance	Phenotypic variance	Heritability
Birth weight	0.05 (0.01)	0.07 (0.01)	0.12 (0.01)	0.21 (0.01)	0.45 (0.01)	0.10 (0.01)
Eight‐week weight	1.09 (0.11)	1.25 (0.08)	3.78 (0.09)	6.33 (0.09)	12.44 (0.08)	0.09 (0.01)
Scan weight	8.97 (0.33)			18.58 (0.24)	27.55 (0.19)	0.33 (0.01)
Muscle depth	1.96 (0.07)			4.59 (0.06)	6.54 (0.05)	0.30 (0.01)
Fat depth	0.28 (0.01)			0.62 (0.01)	0.90 (0.01)	0.31 (0.01)
Footrot	0.04 (0.01)			0.28 (0.01)	0.32 (0.01)	0.12 (0.02)
California Mastitis Test	0.04 (0.02)	0.07 (0.02)		0.40 (0.02)	0.51 (0.01)	0.07 (0.03)

**TABLE 4:** Estimates for genetic (below diagonal) and phenotypic (above diagonal) correlations; estimate followed by standard error in brackets.

Trait	BWT	EWW	SWT	MD	FD	FRT	CMT
Birth weight		0.34 (0.010)	0.31 (0.007)	0.13 (0.007)	0.04 (0.007)	^N^/_E_	^N^/_E_
Eight‐week weight	0.53 (0.079)		0.76 (0.002)	0.42 (0.004)	0.36 (0.004)	^N^/_E_	0.01 (0.024)
Scan weight	0.60 (0.045)	0.95 (0.007)		0.60 (0.003)	0.54 (0.003)	−0.01 (0.020)	−0.01 (0.024)
Muscle depth	0.19 (0.061)	0.61 (0.031)	0.51 (0.019)		0.42 (0.004)	−0.02 (0.020)	0.02 (0.025)
Fat depth	−0.01 (0.063)	0.52 (0.035)	0.48 (0.019)	0.37 (0.023)		−0.03 (0.020)	−0.02 (0.025)
Footrot	^N^/_E_	^N^/_E_	0.07 (0.096)	−0.05 (0.094)	−0.15 (0.096)		0.04 (0.019)
California Mastitis Test	^N^/_E_	0.12 (0.249)	0.10 (0.149)	−0.06 (0.324)	−0.68 (0.368)	0.28 (0.111)	

*Note*: ^N^/_E_ Correlation could not be estimated due to Negative Sum of Squares.

Abbreviations: BWT, birth weight; CMT, California mastitis test; EWW, eight‐week weight; FD, fat depth; FRT, footrot; MD, muscle depth; SWT, scan weight.

### 3.3 Accuracy values

When comparing accuracy values generated from SS‐BLUP to conventional BLUP, there was almost no change in mean accuracy values for the whole population (*n* = 821,692). For BWT, EWW, SWT, MD and FD, the average difference in accuracy was 0; for FRT and CMT, the change was 0.02 and 0.03, respectively. This is due to very high volume of animals included in the prediction, where some of them are not that very well connected with the genotyped population, hence do not benefit from the inclusion of the genotypes in the evaluation. The results also showed that there are some animals whose EBV accuracy may decrease after the inclusion of genotypes although this was only observed for production traits and for animals that were not phenotyped and ungenotyped. The maximum reduction was 0.03 for BWT, EWW and SWT and 0.04 for MD and FD. There was no observed reduction in accuracy value for FTR and CMT. Further analysis revealed that animals that had lower accuracy values following the inclusion of genotypic information were not themselves genotyped or phenotyped and had no close relatives that were phenotyped either. The accuracy for the conventional BLUP EBVs of these animals was less than 0.15 meaning that these animals were not likely to be serious candidates for selection. There were no reductions in accuracy values for any traits for any of the animals that had been genotyped.

The accuracy of GEBVs increased for the majority of genotyped animals compared with their conventional evaluation. The maximum increase in accuracy values were 0.40 for BWT, 0.32 for FD, 0.31 for MD, 0.25 for EWW and 0.22 for SWT. For health traits, these were 0.47 for FRT and 0.52 for CMT. These high increases were observed for animals that were genotyped, but not phenotyped.

**FIGURE 2:**
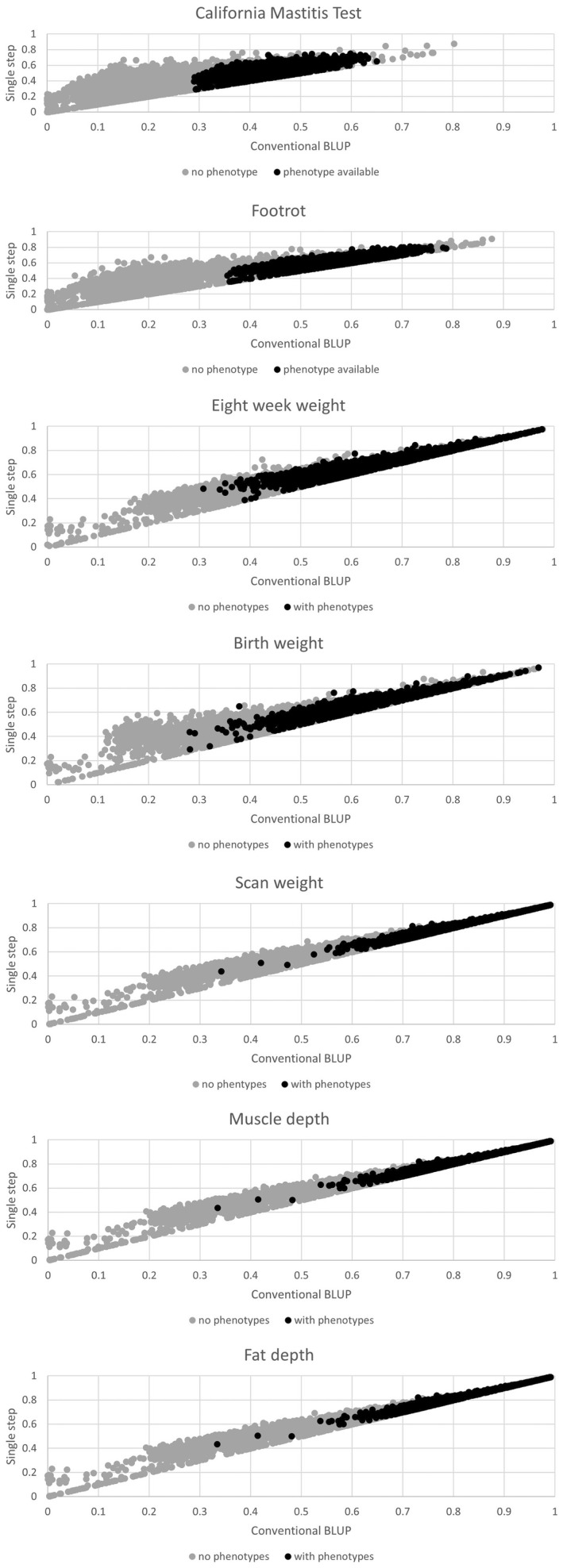
Regression of the accuracy of estimated breeding values derived from single step BLUP on conventional BLUP for genotyped animals with and without phenotypes by trait. Grey dots represent genotyped animals without phenotypes and black dots represent animals with both genotypes and phenotypes available.

Changes in the accuracy of EBV from SS‐BLUP and BLUP for genotyped animals are summarized in Table 5 which shows there are no genotyped animals with reduced accuracy values following the inclusion of their genotypes in the genetic evaluation. On average, the biggest change in accuracy values is observed for traits with the lowest heritability, which are the health traits (CMT and FRT) and 8‐week weight. Changes in accuracy values were greater for animals with no phenotypic information available, meaning that the inclusion of genotypic information is critical to increase the precision of EBV especially for young animals or for males in terms of measuring the CMT. The average changes in accuracy values for un‐phenotyped animals were 170% higher than those from the reference population (animals which are both genotyped and phenotyped). The accuracy values for all traits obtained with conventional BLUP versus SS‐BLUP for both phenotyped and non‐phenotyped animals that were genotyped are shown on Figure 2, illustrating the potential of genomic information to enhance the accuracy values, especially for hard to measure health traits with low heritability (FRT and CMT), where the maximum accuracy increased from 0.18 to 0.47 for FRT and 0.30 to 0.52 for CMT. For traits that are more easily recorded, have more records available and which are moderately heritable (SWT, MD and FD), the increase of accuracy for genotyped animals is still clear but substantially lower than for FRT or CMT. For all traits of this study, phenotyped animals had more accurate EBVs regardless of the evaluation method, which is in accordance with the theoretical expectations (Simm, 1998).

**TABLE 5:** Summary of changes* in accuracy of estimated breeding values (EBV) for genotyped animals with and without phenotypic records per trait.

		BWT	EWW	SWT	MD	FD	FRT	CMT
No. animals without phenotype		7223	4305	3784	3878	3887	5612	6482
EBV accuracy change	Avg	0.09	0.09	0.07	0.07	0.07	0.19	0.21
	Min	0.00	0.00	0.00	0.00	0.00	0.00	0.00
	Max	0.40	0.30	0.22	0.22	0.22	0.47	0.52
	*SD*	0.06	0.05	0.04	0.04	0.04	0.09	0.09
No. animals with phenotype		2158	5086	5607	5513	5504	3779	2909
EBV accuracy change	Avg	0.05	0.04	0.02	0.02	0.02	0.06	0.11
	Min	0.00	0.00	0.00	0.00	0.00	0.00	0.00
	Max	0.27	0.18	0.10	0.10	0.10	0.18	0.30
	*SD*	0.03	0.03	0.01	0.01	0.01	0.03	0.05

Abbreviations: Avg, average; BWT, birth weight; CMT, California mastitis test; EWW, eight‐week weight; FD, fat depth; FRT, footrot; Max, maximum; MD, muscle depth; Min, minimum; *SD*, standard deviation; SWT, scan weight.

^*^ Calculated as the SS‐BLUP accuracy minus the conventional BLUP accuracy.

These findings are in line with results from previous studies, demonstrating increased accuracy when information from genotypes is included, such as for Manech Tête Rousse dairy sheep (Macedo et al., 2020), small population of Dorper sheep (Moghaddar et al., 2022) or chicken mortality (Bermann et al., 2020). Furthermore, several studies comparing accuracies obtained from various genomic models indicate single‐step BLUP as the best way for getting high accuracy values in sheep (Baloche et al., 2013) or dairy goats (Mucha, Bunger, & Conington, 2015; Mucha, Mrode, et al., 2015).

## 4 CONCLUSION

This study has combined production and health traits recorded in a well‐phenotyped population of UK Texel sheep. This was the first study to estimate the potential gain in prediction accuracy of adding genomic data into the estimation of breeding values for UK sheep. It has showed that the structure of the data used for the evaluations affects the changes seen in accuracy values, which also differ according to the traits analysed. In all scenarios, adding animal genotypes in a single‐step BLUP evaluation increased the accuracy of prediction compared with conventional BLUP. Therefore, increased animal genotyping is recommended in a breeding programme in order to improve the accuracy of estimated breeding values and reduce the risks associated with making selection decisions. It also will achieve accelerated rates of genetic gain, enhanced efficiency of production and lead to enhanced animal welfare when health traits are included in the breeding programme.

## AUTHOR CONTRIBUTIONS

KK undertook the analysis and wrote the paper. JC and GB were principal investigators (PI) for the original research project, collated the data and wrote the paper. SM, JY and ES reviewed and wrote the manuscript. ES also collected the data. All authors have read and approved the final manuscript.

## FUNDING INFORMATION

This work was supported by the InnovateUK/(BBSRC) project reference number 131791 (BB/M018377/1) and project reference number 102646 (BB/M02833X/1) and Horizon 2020 Research and Innovation Programme under the grant agreement No. 772787 (SMARTER).

## Data Availability

The data that support the study findings are private and confidential.

## References

[jbg12771-bib-0001] Auvray, B. , McEwan, J. C. , Newman, S.‐A. , Lee, M. , & Dodds, K. (2014). Genomic prediction of breeding values in the New Zealand sheep industry using a 50 K SNP chip. Journal of Animal Science, 92, 4375–4389. 10.2527/jas.2014-7801 25149326

[jbg12771-bib-0002] Baloche, G. , Legarra, A. , Sallé, G. , Larroque, H. , Astruc, J.‐M. , Robert‐Granié, C. , & Barillet, F. (2013). Assessment of accuracy of genomic prediction for French Lacaune dairy sheep. Journal of Dairy Science, 97, 1107–1116. 10.3168/jds.2013-7135 24315320

[jbg12771-bib-0003] Bermann, M. , Legarra, A. , Hollifield, M. K. , Masuda, Y. , Lourenco, D. , & Misztal, I. (2020). Validation of single‐step GBLUP genomic predictions from threshold models using the linear regression method: An application in chicken mortality. Journal of Animal Breeding and Genetics, 138(1), 4–13. 10.1111/jbg.12507 32985749 PMC7756448

[jbg12771-bib-0004] Berry, D. P. , Dunne, F. L. , McHugh, N. , McParland, S. , O'Brien, A. C. , & Twomey, A. J. (2022). The development of effective ruminant breeding programmes in Ireland from science to practice. Irish Journal of Agricultural and Food Research, 61(1), 38–54. 10.15212/ijafr-2020-0149

[jbg12771-bib-0005] Berry, D. P. , Kearney, F. , Evans, R. , Wall, E. , & Cromie, A. (2016). S0116 genomic evaluations in dairy cattle, beef cattle, and sheep in Ireland. Journal of Animal Science, 94(4), 8–9. 10.2527/jas2016.94supplement48a

[jbg12771-bib-0037] Christensen, O. F. (2012). Compatibility of pedigree‐based and marker‐based relationship matrices for single‐step genetic evaluation. Genetics Selection Evolution, 44, 37. 10.1186/1297-9686-44-37 PMC3549765 23206367

[jbg12771-bib-0006] Christensen, O. F. , & Lund, M. S. (2010). Genomic prediction when some animals are not genotyped. Genetics Selection Evolution, 42, 2. 10.1186/1297-9686-42-2 PMC2834608 20105297

[jbg12771-bib-0007] Conington, J. , Hosie, B. , Nieuwhof, G. J. , Bishop, S. C. , & Bünger, L. (2008). Breeding for resistance to footrot‐the use of hoof lesion scoring to quantify footrot in sheep. Veterinary Research Communications, 32(8), 583–589. 10.1007/s11259-008-9062-x 18478350

[jbg12771-bib-0008] Fitzmaurice, S. , Conington, J. , McHugh, N. , & Banos, G. (2021). Towards future genetic evaluations for live weight and carcass composition traits in UK sheep. Small Ruminant Research, 196, 106327. 10.1016/j.smallrumres.2021.106327

[jbg12771-bib-0009] Gilmour, A. R. , Gogel, B. J. , Cullis, B. R. , Welham, S. J. , & Thompson, R. (2015). ASRemlUserGuideRelease 4.1. Structural Specification. Available at www.vsni.co.uk

[jbg12771-bib-0010] Hayes, B. J. (2011). Technical note: Efficient parentage assignment and pedigree reconstruction with dense single nucleotide polymorphism data. Journal of Dairy Science, 94(4), 2114–2117. 10.3168/jds.2010-3896 21427003

[jbg12771-bib-0011] Hayes, B. J. , Bowman, P. J. , Daetwyler, H. D. , Kijas, J. W. , & van der Werf, J. H. (2012). Accuracy of genotype imputation in sheep breeds. Animal Genetics, 43(1), 72–80. 10.1111/j.1365-2052.2011.02208.x 22221027

[jbg12771-bib-0012] Henderson, C. R. (1949). Estimation of changes in herd environment. Journal of Dairy Science, 32, 709 (Abstract).

[jbg12771-bib-0013] Kaseja, K. , Mucha, S. , Yates, J. , Smith, E. , Banos, G. , & Conington, J. (2022). Discovery of hidden pedigree errors combining genomic information with the genomic relationship matrix in Texel sheep. Animal, 16(3), 100468. 10.1016/j.animal.2022.100468 35190320

[jbg12771-bib-0014] Lambe, N. R. , Conington, J. , Bishop, S. C. , McLean, K. A. , Bünger, L. , McLaren, A. , & Simm, G. (2008). Relationships between lamb carcass quality traits measured by X‐ray computed tomography and current UK hill sheep breeding goals. Animal, 2(1), 36–43. 10.1017/S1751731107001061 22444961

[jbg12771-bib-0015] Legarra, A. , Aguilar, I. , & Misztal, I. (2009). A relationship matrix including full pedigree and genomic information. Journal of Dairy Science, 92(9), 4656–4663. 10.3168/jds.2009-2061 19700729

[jbg12771-bib-0016] Lidauer, M. , Matilainen, K. , Mäntysaari, E. , Pitkänen, T. , Taskinen, M. , & Strandén, I. (2015). MiX99: Technical reference guide for MiX99 solver. Available at https://www.luke.fi

[jbg12771-bib-0017] Macciotta, N. P. P. , Gaspa, G. , Steri, R. , Nicolazzi, E. L. , Dimauro, C. , Piermati, C. , & Cappio‐Borlino, A. (2010). Using eigenvalues as variance priors in the prediction of genomic breeding values by principal component analysis. Journal of Dairy Science, 93(6), 2765–2774. 10.3168/jds.2009-3029 20494186

[jbg12771-bib-0018] Macedo, F. L. , Christensen, O. F. , Astruc, J. M. , Aguilar, I. , Masuda, Y. , & Legarra, A. (2020). Bias and accuracy of dairy sheep evaluations using BLUP and SSGBLUP with metafounders and unknown parent groups. Genetics, Selection, Evolution, 52, 47. 10.1186/s12711-020-00567-1 PMC7425573 32787772

[jbg12771-bib-0019] McLaren, A. , Kaseja, K. , Yates, J. , Mucha, S. , Lambe, N. , & Conington, J. (2018). New mastitis phenotypes suitable for genomic selection in meat sheep and their genetic relationships with udder conformation and lamb live weights. Animal, 12(12), 2470–2479. 10.1017/S1751731118000393 29576020

[jbg12771-bib-0020] Misztal, I. , Legarra, A. , & Aguilar, I. (2009). Computing procedures for genetic evaluation including phenotypic, full pedigree, and genomic information. Journal of Dairy Science, 92(9), 4648–4655. 10.3168/jds.2009-2064 19700728

[jbg12771-bib-0021] Misztal, I. , & Wiggans, G. R. (1988). Approximation of prediction error variance in large‐scale animal models. Journal of Dairy Science, 71(2), 27–32. 10.1016/S0022-0302(88)79976-2

[jbg12771-bib-0022] Moghaddar, N. , Brown, D. J. , Swan, A. A. , Gurman, P. M. , Li, L. , & van der Werf, J. H. (2022). Genomic prediction in a numerically small breed population using prioritized genetic markers from whole‐genome sequence data. Journal of Animal Breeding and Genetics, 139(1), 71–83. 10.1111/jbg.12638 34374454

[jbg12771-bib-0023] Mortimer, S. I. , Fogarty, N. M. , van der Werf, J. H. J. , Brown, D. J. , Swan, A. A. , Jacob, R. H. , Geesink, G. H. , Hopkins, D. L. , Hocking Edwards, J. E. , Ponnampalam, E. N. , Warner, R. D. , Pearce, K. L. , & Pethick, D. W. (2018). Genetic correlations between meat quality traits and growth and carcass traits in merino sheep. Journal of Animal Science, 6(9), 3582–3598. 10.1093/jas/sky232 PMC6127828 29893862

[jbg12771-bib-0024] Mucha, S. , Bunger, L. , & Conington, J. (2015). Genome‐wide association study of footrot in Texel sheep. Genetics Selection Evolution, 47, 35. 10.1186/s12711-015-0119-3 PMC4415250 25926335

[jbg12771-bib-0025] Mucha, S. , Mrode, R. , MacLaren‐Lee, I. , Coffey, M. , & Conington, J. (2015). Estimation of genomic breeding values for Milk yield in UK dairy goats. Journal of Dairy Science, 98(11), 8201–8208. 10.3168/jds.2015-9682 26342984

[jbg12771-bib-0026] Pacheco, A. , McNeilly, T. N. , Banos, G. , & Conington, J. (2021). Genetic parameters of animal traits associated with coccidian and nematode parasite load and growth in Scottish blackface sheep. Animal, 15(4), 100185. 10.1016/j.animal.2021.100185 33653675

[jbg12771-bib-0027] Palhière, I. , Gousseau, V. , & Colleau, J. J. (2022). Implementing genomic management of diversity and selection in French dairy goats. Proceedings, 12th world congress of genetics applied to livestock production.

[jbg12771-bib-0028] R Core Team . (2022). R: A language and environment for statistical computing. R foundation for statistical computing. Available at https://www.R‐project.org/

[jbg12771-bib-0029] Rupp, R. , Mucha, S. , Larroque, H. , McEwan, J. , & Conington, J. (2016). Genomic application in sheep and goat breeding. Animal Frontiers, 6(1), 39–44. 10.2527/af.2016-0006

[jbg12771-bib-0030] Safari, E. , Fogarty, N. M. , & Gilmour, A. R. (2005). A review of genetic parameter estimates for wool, growth, meat and reproduction traits in sheep. Livestock Production Science, 92(3), 271–289. 10.1016/j.livprodsci.2004.09.003

[jbg12771-bib-0031] Sheep Ireland Guide & Directory of Breeders (2020). Available at https://www.sheep.ie/wp‐content/uploads/2020/08/SHEEP‐IRELAND‐DIRECTORY‐BOOK‐2020‐rev9‐002.pdf

[jbg12771-bib-0032] Simm, G. (1998). Genetic Improvement of Cattle and Sheep. Farming Press distributed by CABI Publishing, Wallingford (United Kingdom) and New York. ISBN: 0‐85236‐351‐6.

[jbg12771-bib-0033] Teissier, M. , Brito, L. F. , Schenkel, F. , Bruni, G. , Fresi, P. , Bapst, B. , RobertGranié, C. , & Larroque, H. (2022). Genetic characterization and connectedness of dairy goats in Canada, France, Italy and Switzerland. Proceedings, 12th world congress of genetics applied to livestock production.

[jbg12771-bib-0034] VanRaden, P. M. (2008). Efficient Methods to compute genomic predictions. Journal of Dairy Science, 91(11), 4414–4423. 10.3168/jds.2007-0980 18946147

[jbg12771-bib-0035] VanRaden, P. M. , O'Connell, J. R. , Wiggans, G. R. , & Weigel, K. A. (2011). Genomic evaluations with many more genotypes. Genetics Selection Evolution, 43(1), 1–11. 10.1186/1297-9686-43-10 PMC3056758 21366914

[jbg12771-bib-0036] Walkom, S. F. , Bunter, K. L. , Raadsma, H. W. , Gurman, P. M. , Brown, D. J. , Gibson, W. , Wilding, E. , & Ferguson, M. B. (2022). Development of breeding values for susceptibility to virulent footrot in sheep: A strategy to accommodate variable disease progression at time of scoring. Animal, 16(5), 100514. 10.1016/j.animal.2022.100514 35421686

